# Comprehensive genomic and transcriptomic characterization of high-grade gastro-entero-pancreatic neoplasms

**DOI:** 10.1038/s41416-024-02705-8

**Published:** 2024-05-10

**Authors:** Valentina Angerilli, Giovanna Sabella, Michele Simbolo, Vincenzo Lagano, Giovanni Centonze, Marco Gentili, Alessandro Mangogna, Jorgelina Coppa, Giada Munari, Gianluca Businello, Chiara Borga, Francesca Schiavi, Sara Pusceddu, Rita Leporati, Simone Oldani, Matteo Fassan, Massimo Milione

**Affiliations:** 1 Department of Medicine (DIMED), University of Padua, Padua, Italy; 2 First Pathology Unit, Department of Pathology and Laboratory Medicine, Fondazione IRCCS Istituto Nazionale dei Tumori, Milan, Italy; 3 Department of Diagnostics and Public Health, University and Hospital Trust of Verona, Verona, Italy; 4 Institute of Pathological Anatomy, Department of Medicine (DMED), University of Udine, Udine, Italy; 5 Hepatology and Hepato-Pancreatic-Biliary Surgery and Liver Transplantation, Fondazione IRCCS Istituto Nazionale Tumori, Milan, Italy; 6 Veneto Institute of Oncology (IOV-IRCCS), Padua, Italy; 7 Medical Oncology and Hematology Department, Fondazione IRCCS Istituto Nazionale Tumori, Milan, Italy

**Keywords:** Oncology, Surgical oncology, Molecular medicine

## Abstract

**Background:**

High-grade gastro-entero-pancreatic neoplasms (HG GEP-NENs) can be stratified according to their morphology and Ki-67 values into three prognostic classes: neuroendocrine tumors grade 3 (NETs G3), neuroendocrine carcinomas with Ki-67 < 55% (NECs <55) and NECs with Ki-67 ≥ 55% (NECs ≥55).

**Methods:**

We analyzed a cohort of 49 HG GEP-NENs by targeted Next-Generation Sequencing (TrueSight Oncology 500), RNA-seq, and immunohistochemistry for p53, Rb1, SSTR-2A, and PD-L1.

**Results:**

Frequent genomic alterations affected *TP53* (26%), *APC* (20%), *KRAS* and *MEN1* (both 11%) genes. NET G3 were enriched in *MEN1* (*p* = 0.02) mutations, while both NECs groups were enriched in *TP53* (*p* = 0.001), *APC* (*p* = 0.002) and *KRAS* (*p* = 0.02) mutations and tumors with TMB ≥ 10 muts/Mb (*p* = 0.01). No differentially expressed (DE) gene was found between NECs <55% and NECs ≥55%, while 1129 DE genes were identified between NET G3 and NECs. A slight enrichment of CD4^+^ and CD8^+^ T cells in NECs and of cancer-associated fibroblasts and macrophages (M2-like) in NET G3. Multivariate analysis identified histologic type and Rb1 loss as independent prognostic factors for overall survival.

**Conclusions:**

This study showed that GEP-NET G3 and GEP-NECs exhibit clear genomic and transcriptomic differences, differently from GEP-NECs <55% and GEP-NECs ≥55%, and provided molecular findings with prognostic and potentially predictive value.

## Background

Gastro-entero-pancreatic neuroendocrine neoplasms (GEP-NENs) are a heterogeneous group of malignancies with neuroendocrine differentiation. According to WHO 2019 [1] and 2022 [2] criteria, GEP-NENs are currently classified as: i) neuroendocrine tumors (NETs), ii) neuroendocrine carcinomas (NECs), and iii) mixed non-neuroendocrine-neuroendocrine neoplasms. GEP-NETs should be graded on the basis of Ki-67 proliferation index.

International clinical guidelines [3, 4] recognize GEP-NET G3 and GEP-NECs as a common overarching concept called High-Grade Gastro-entero-pancreatic Neuroendocrine Neoplasms (HG GEP-NENs), whilst acknowledging the importance of the distinction between GEP-NET G3 and GEP-NECs for prognostic and therapeutic purposes. GEP-NECs have a poor prognosis with a median overall survival (OS) < 1 year in advanced, treated cases and the treatment of choice is platinum-based chemotherapy (PBC) [5, 6]; GEP-NETs G3 have better survival outcomes than GEP-NECs, tend to metastasize early, and are treated with systemic non-PBC [7].

Milione et al. [8] identified and validated three HG GEP-NENs subgroups with prognostic relevance: well-differentiated neoplasms with Ki-67 proliferative index ≥20%, and poorly differentiated GEP-NENs with Ki-67 index <55% or ≥55%. Moreover, results from the NORDIC NEC study [5] suggested that Ki-67 index of 55% may be a useful cut-off to better stratify HG GEP-NEN patients in terms of response to PBC and OS. Other studies demonstrated the prognostic value of Ki-67 with a cut-off of 55 in HG GEP-NENs [7, 9, 10]. Conversely, Elvebakken et al. confirmed that GEP-NEC with Ki-67 ≥ 55% had a significantly better response rate to chemotherapy compared to GEP-NECs with Ki-67 < 55%, but no difference in OS between GEP-NECs <55% and GEP-NECs ≥55% was found [11]. The prognostic role of Ki-67 labeling index ≥55% has been included in ENETS guidelines since 2016 [3, 12], thus recognizing it as a powerful tool for patient stratification in GEP-NENs. Moreover, there is increasing evidence that alkylating-based chemotherapy should be considered for GEP-NECs <55% [13].

GEP-NECs usually harbor *TP53* or *RB1* mutations, while *MEN1*, *DAXX*, and *ATRX* mutations are distinctive for well-differentiated pancreatic GEP-NETs [14]. A recent study by Puccini et al. [15] has shown that HG GEP-NENs and low-grade (LG) GEP-NENs are molecularly distinct entities. In fact, *TP53* and *RB1* as well as *KRAS*, *APC*, *BRAF*, and *PI3KCA* were frequently mutated in HG but not in LG GEP-NENs. However, unlike GEP-NECs, GEP-NETs G3 retain the mutation profile of other well-differentiated NETs, with frequent mutations in *MEN1*, *ATRX*, *DAXX*, *SETD2* and *TP53* [16]. According to Hijioka et al. [17], *KRAS* mutations and Rb loss are favorable predictors of response to PBC in pancreatic HG NENs. On the other hand, Elvebakken et al. found no predictive role of *KRAS* mutations in treatment effect in a cohort of HG GEP-NENs [18]. Additionally, NECs with microsatellite instability (MSI) have been reported to have a better prognosis than their microsatellite stable (MSS) counterpart [19, 20].

As for transcriptomic profiling, no study has focused on HG GEP-NENs until now. Yachida et al. performed RNA-seq on a cohort of 115 GEP-NENs [21]. Transcription factors for neuroendocrine differentiation, especially the *SOX2* gene, appeared overexpressed in most gastrointestinal NECs.

To date, the classification of HG GEP-NENs by morphology and proliferation rate remains the most reliable method for patient prognostication and therapeutic stratification based on response to chemotherapy [11]. However, HG GEP-NENs therapeutic approach is still lacking effective targeted treatments and reliable biomarkers to stratify patient prognosis and response to standard chemotherapy.

## Methods

### Patients and samples

This study was performed according to the clinical standards of the 1975 and 1983 Declaration of Helsinki and was approved by the Ethical Committee of Fondazione IRCCS Istituto Nazionale dei Tumori (INT), Milan, Italy (n° INT 21/16). All patients had signed an informed consent for the use of their data for research purposes.

Between 2010 and 2020, the surgical pathology and clinical databases of INT (an Excellence Centre for the therapy of NENs) were retrospectively searched and patients with one of the following diagnoses were selected: “neuroendocrine neoplasm”, “NET” and “NEC” (Supplementary information). Adopted exclusion criteria were: i) cases with mixed neuroendocrine and non-neuroendocrine components; ii) cases with inadequate material for NGS analysis; iii) not GEP origin.

Selected cases were studied applying tumor grading according to WHO 2019 [1] and WHO 2022 [2], and tumor staging (TNM) according to the Union for International Cancer Control/American Joint Committee on Cancer (8th edition was applied UICC/AJCC) [22].

Thus, a series of 49 formalin-fixed and paraffin-embedded (FFPE) HG NENs samples was included in the present study. All the samples were surgical resection specimens.

### Immunohistochemistry (IHC)

The immunohistochemical study included the detection of the following markers: a) Chromogranin-A and Synaptophysin (general neuroendocrine markers) to confirm the neuroendocrine differentiation; b) Ki-67 labeling index calculation, using the MIB antibody and expressed as a percentage of at least 500 cells counted in areas of strongest nuclear labeling (hot spots) as suggested in the gastroenteropancreatic neuroendocrine tumor WHO 2019 [1] and 2022 [2] guidelines; c) Rb1 was considered positive regardless of the number of positive cells and negative in case of loss in all tumor cells; d) p53 was considered aberrant in the presence of complete loss of nuclear expression in tumor cells with positive stromal cells as internal control (i.e., null phenotype) or overexpressed if strong nuclear expression was present in >20% of tumor cells; p53 heterogenous expression was considered as normal [23, 24]; e) Somatostatin receptor 2A (SSTR-2A) assessed according to Volante et al. [25] (positive: 2+, 3+; negative: 0, 1+ score); f) PD-L1 was evaluated separately in neoplastic cells and in intra-tumor lymphocytes.

IHC was performed using the antibodies listed in Supplementary Table S1. IHC slides were jointly evaluated by three pathologists (MM, VA, and GS).

### Tissue collection and nucleic acid isolation

Five 10-µm paraffin embedded sections were used to extract the DNA, using the QIAmpFFPE tissue Kit (Qiagen) according to the manufacturer’s instructions. Quantification of extracted DNA was done using the Qubit® 3.0 fluorometer and the Qubit® DNA BR Assay kit (Thermo Fisher Scientific). An equal number of sections for the same sample were used to extract the total RNA, using the ReliaPrep™ RNA Tissue Miniprep System (Promega) according to the manufacturer’s instructions. Quantification of extracted RNA was done using the Qubit® 3.0 fluorometer and the Qubit® RNA HS Assay kit (Thermo Fisher Scientific).

### Genomic profiling by TSO500

120 ng of DNA and 200 ng of RNA were used for TruSight Oncology 500 (TSO500). DNA fragmentation was performed using the Covaris (Woburn, MA) M220 focused-ultrasonicator. The library was prepared manually according to the manufacturer’s protocol. The Qubit dsDNA HS Assay Kit (Thermo Fisher Scientific) was used to quantify the hybridization capture-enriched NGS libraries before library normalization. NGS sequencing was performed on a NextSeq 550 instrument (Illumina) with eight DNA and eight cDNA libraries per sequencing run. The manufacturer’s quality control criteria were used to determine whether result was valid, median insert size ≥70 bp, median exon coverage ≥100 count, and percentage of exons with coverage of at least 100 count ≥90%. For the analysis of alterations (single nucleotide variants [SNVs], deletions, insertions, and fusions), tumor mutational burden (TMB) and percentage of MSI the Pierian platform was used.

### Mutational signature analysis

The contribution of COSMIC mutational signatures in targeted sequencing data was calculated using the deconstructSigs R package. Cases with a small number of mutations (<50) were excluded from deconstructSigs analysis. Clustering by contribution of mutational signatures was performed using unsupervised hierarchical clustering with cosine distance and Ward linkage.

### RNA-seq gene-expression profiling

Briefly, 50 ng of total RNA have been used to create the libraries, accordingly to the manufacturer’s instructions SMARTer Stranded Total RNA-Seq Kit v3 - Pico Input Mammalian. The kit features a workflow that retains strand information and incorporates indexes and adapters during the reverse-transcription and PCR-amplification steps. Libraries were quantified using the Qubit 4.0 fluorometer (Thermo Fisher Scientific, Milan, Italy) and quality was checked using 4200 Tape Station (Agilent) and pooled to equimolar concentration. The Next-generation sequencing (NGS) was performed on a NovaSeq Platform (Illumina).

### Data processing and bioinformatics analysis – RNA

Quality check of the raw sequencing reads was performed using FastQC (v0.11.9). Quality filtering and check of the reads was performed using Fastp (v0.20.1) with the following set of parameters: length_required = 36, cut_right, cut_right_window_size = 4, cut_right_mean_quality = 15, trim_poly_g, overrepresentation_analysis.

The alignment of high-quality reads on the reference human genome GRCh38 (Gencode release 37) was performed using STAR (v2.7.8a). The quality control of the alignments was performed using RSeQC v4.0.0.

Counts were normalized and transformed using the “DESeq2” package for R [26]. The expression data were subjected to quality control using the workflow defined by Law et al. [27]. Visualization and clustering were performed using the “ComplexHeatmap” package for R [28].

A sample map was obtained using the Uniform Manifold Approximation and Projection (UMAP) method on the genes with the most variable expression genes (genes considered explained the 70% of the total variance). UMAP is a dimensionality reduction method based on manifold learning techniques, which are adapted to non-linear data in contrast with the commonly used principal component analysis (PCA) method. First, it builds a topological representation of the high-dimensional data, and second, it finds the best low-dimensional representation of this topological structure [29]. UMAP representations were generated using the umap function from the R package umap (v. 0.2.5.0)[Konopka T (2019) umap: Uniform Manifold Approximation and Projection]. All the parameters were set to their default values except then neighbors’ parameter. This parameter defines the number of neighbors considered to learn the structure of the topological space. Varying this parameter from small to large values enables the user to find a trade-off between local and global preservation of the space, respectively. We built the sample map by setting the n_neighbors parameter to the total number of samples.

Differential expression analysis between groups was performed using Deseq2 algorithm. A gene was considered differentially expressed if it showed an adjusted P-value under 0.05. Cancer and biological signatures were retrieved from Hallmark pathways and C2 signatures of MSigDB and the cluster-specific enriched gene sets using the normalized count matrix was determined. We applied GSEA using GAGE [30] R package between clusters to get pairwise significant up and down-regulated pathways. An approach based on the ssGSEA score was used for determining the signatures differently enriched between all the clusters. We performed a z-score normalization of the pathway scores in the clusters. A positive correlation between the sample and the specific pathway is represented by a z-score >0. We considered only the differently related pathways (*p*-value <0.05 according to Benjamini-Hochberg test). All samples were grouped according to their molecular class.

### Statistics

Data were analyzed by descriptive statistics. Associations between clinicopathological and molecular features and GEP-NETs G3, GEP-NECs with Ki-67 index <55% (GEP-NECs <55%) and GEP-NEC with Ki-67 index ≥55% (GEP-NECs ≥55%) were assessed using the Fisher exact test for categorical variables and Kruskal–Wallis test for continuous variables. OS was assessed from the date of diagnosis to the date of death or last follow-up. Progression-free survival (PFS) of chemotherapy was assessed from the start of the therapy to the date of first relapse, death, or last follow-up, whichever occurred first. OS and PFS curves were drawn using the Kaplan–Meier method. The log-rank test was used to assess the survival difference between patients’ groups. Univariable and multivariable Cox proportional regression models were used to assess the association between clinicopathological characteristics and OS. Hazard ratios (HR) are presented with respective 95% confidence interval (CI). Data analysis was performed using the R environment for statistical computing and graphics (R Foundation, Vienna, Austria - Version 4.0.3). All tests were two-sided and *p*-values < 0.05 were considered statistically significant.

## Results

### Patient and tumor characteristics

Among the 49 HG GEP NENs included in this study, 21 (42.9%) were GEP-NETs G3, 12 (24.5%) GEP-NECs <55% and 16 (32.6%) GEP-NECs ≥55%. The most frequent primary sites were pancreas (44.9%), colon-rectum (24.4%), and stomach (14.3%). In our cohort, the GEP-NETs G3 group was enriched in primitive pancreatic tumors (14/21, *p* = 0.02).

The median age of the patients was 61 (range 21–87) and the male-to-female *ratio* was 1.7. Most patients (89.4%) received adjuvant systemic therapy, including 30 patients (63.8%) who received chemotherapy. None of the patients received neoadjuvant treatment. Overall, 40 primary tumor samples and 9 metastatic site samples were included. Most tumors (63.8%) were diagnosed at stage IV.

The clinico-pathologic features of the cohort according to morphology and Ki-67 are reported in Table 1, whereas according to WHO class in Supplementary Table S2.

**Table 1:** Characteristics of patients with high-grade neuroendocrine neoplasms according to morphology and Ki-67

	All patients	NET G3	NEC< 55%	NEC≥ 55%	*p*-value^a^	*p*-value^b^
Total	49 (100)	21 (100)	12 (100)	16 (100)		
Gender						
Female	18 (36.7)	8 (38.1)	6 (50.0)	4 (25.0)		
Male	31 (63.3)	13 (61.9)	6 (50.0)	12 (75.0)	0.4	0.2
Age						
Mendian [range]	61 [21–87]	58 [21–80]	59 [26–87]	66 [39–76]	0.4	0.2
Stage						
I	1 (2.1)	1 (4.8)	0 (0.0)	0 (0.0)		
II	3 (6.4)	0 (0.0)	2 (18.2)	1 (6.7)		
III	13 (27.7)	6 (28.6)	3 (27.3)	4 (26.7)		
IV	30 (63.8)	14 (66.7)	6 (54.6)	10 (66.7)	0.6	0.8
Site Origin						
Colorectal	12 (24.5)	2 (9.5)	4 (33.3)	6 (37.5)		
Gastroesophageal	9 (18.4)	2 (9.5)	2 (16.7)	5 (31.3)		
Ileum-duodenum-gallbladder	6 (12.2)	3 (14.3)	1 (8.3)	2 (12.5)		
Pancreas	22 (44.9)	14 (66.7)	5 (41.7)	3 (18.7)	0.07	0.7
Site Primitive						
Pancreas	22 (44.9)	14 (66.7)	5 (41.7)	3 (18.8)		
Other	27 (55.1)	7 (33.3)	7 (58.3)	13 (81.2)	**0.02**	0.2
Therapy						
None	5 (10.6)	3 (14.3)	1 (9.1)	1 (6.7)		
Chemotherapy	30 (63.8)	10 (47.6)	7 (63.6)	13 (86.7)		
Others	3 (6.4)	2 (9.5)	1 (9.1)	0 (0.0)		
SSA	9 (19.1)	6 (28.6)	2 (18.2)	1 (6.7)	0.4	0.5
*Molecular Features*						
TMB						
Mendian [range]	4.0 [0.8–79.3]	3.9 [0.8–14.9]	3.9 [0.8–11.2]	7.5 [1.6–79.3]	0.07	0.1
TMB						
<10	35 (81.4)	19 (95.0)	8 (88.9)	8 (57.1)		
>10	8 (18.6)	1 (5.0)	1 (11.1)	6 (42.9)	**0.01**	0.2
*TP53*						
WT	34 (73.9)	20 (95.2)	8 (72.7)	6 (42.9)		
Mutated	12 (26.1)	1 (4.8)	3 (27.3)	8 (57.1)	**0.001**	0.2
*APC*						
WT	37 (80.4)	21 (100.0)	7 (63.6)	9 (64.3)		
Mutated	9 (19.6)	0 (0.0)	4 (36.4)	5 (35.7)	**0.002**	1.0
*KRAS*						
WT	41 (89.1)	21 (100.0)	10 (90.9)	10 (71.4)		
Mutated	5 (10.9)	0 (0.0)	1 (9.1)	4 (28.6)	**0.02**	0.3
*MEN1*						
WT	41 (89.1)	16 (76.2)	11 (100.0)	14 (100.0)		
Mutated	5 (10.9)	5 (23.8)	0 (0.0)	0 (0.0)	0.06	–
*Immunohistochemical Markers*						
p53						
Heterogeneus	22 (44.9)	12 (57.1)	5 (41.7)	5 (31.3)		
Overexpressed or absent	27 (55.1)	9 (42.9)	7 (58.3)	11 (68.7)	0.28	**0.04**
Rb1						
Absent	10 (20.4)	1 (4.8)	1 (8.3)	8 (50.0)		
Present	39 (79.6)	20 (95.2)	11 (91.7)	8 (50.0)	**0.002**	**0.04**
SSTR-2A						
Absent (0-1)	23 (46.9)	6 (28.6)	7 (58.3)	10 (62.5)		
Present (2–3)	26 (53.1)	15 (71.4)	5 (41.7)	6 (37.5)	0.09	1.0

The values in bold corresponding to the columns (*p*-values) indicate statistically significant values (i.e. < 0.05).

*NET* neuroendocrine tumor, *NEC* neuroendocrine carcinoma, *SSA* somatostatin analogues, *TBM* tumor mutational burden, *TP53* tumor protein 53 gene, *WT* wild type, *APC* adenomatous polyposis coli gene, *KRAS* Kirsten rat sarcoma virus gene, *MEN1* multiple endocrine neoplasia type 1 gene, *p53* tumor suppressor p53, *Rb1* retinoblastoma-associated protein, *SSTR-2A* somatostatin receptor 2 A.

^a^*p*-value based on the Fisher’s exact for categorical variables or Kruskal–Wallis test for continuous variables.

^b^*p*-value evaluated in NEC only.

### Immunohistochemical analysis

All 49 samples were subjected to IHC analysis. Aberrant p53 expression was observed in 55.1% of tumors; either complete loss (38.8%) or overexpression (16.3%). Aberrant p53 expression was more frequent in GEP-NECs ≥55%, followed by GEP-NET G3 and GEP-NECs <55% (42.9% vs 58.3% vs 68.7%), without reaching statistical significance.

Rb1 loss was detected in 50.0% of GEP-NECs ≥55%, and in 8.3% of GEP-NECs <55% and 4.8% of GEP-NET G3 (*p* = 0.002).

SSTR-2A expression (score 2-3) was higher in GEP-NET G3 (71.4%), than in GEP-NECs ≥55% (41.7%) and GEP-NECs <55% (37.5%), without reaching statistical significance (*p* = 0.09).

When comparing primary tumor sites, HG colorectal NENs had a higher frequency of tumors with Rb1 loss (50.0%), followed by HG gastroesophageal NENs (30.0%) (*p* = 0.03). Colorectal and pancreatic NECs were enriched in p53 aberrant tumors (*p* = 0.004).

PD-L1 expression was absent both in neoplastic cells and in intra-tumor lymphocytes in all cases of our cohort.

**Fig. 1:**
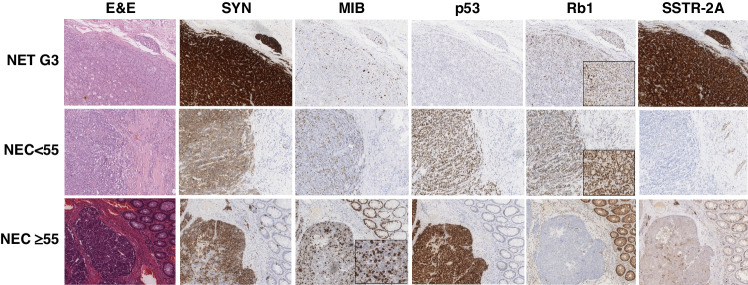
Morphological and immunohistochemical features of three selected cases of GEP-NET G3, GEP-NEC < 55% and GEP-NEC ≥ 55%. SYN and MIB are used to prove the neuroendocrine differentiation and discriminate between GEP-NEC < 55% and GEP-NEC ≥ 55%. p53 is aberrant (overexpression) in GEP-NEC < 55% and GEP-NEC ≥ 55%, Rb1 is lost in the GEP-NEC ≥ 55%, and SSTR2A expression is present in the GEP-NET G3. GEP gastroenteropancreatic, NET neuroendocrine tumor, NEC neuroendocrine carcinoma; NEC < 55%, NEC with Ki-67 < 55%; NEC ≥ 55%, NEC with Ki-67 ≥ 55%, E&E hematoxylin and eosin, SYN synaptophysin, MIB antibody for detecting the Ki-67 antigen, p53 tumor suppressor p53, Rb1 retinoblastoma-associated protein, SSTR-2A somatostatin receptor 2A.

The immunohistochemical features of the cohort according to morphology and Ki-67 are reported in Table 1 and Fig. 1.

### Genomic profile of HG GEP-NENs

The molecular features of the cohort according to morphology and Ki-67 indexes are reported in Table 1. The clinico-pathologic and molecular features of our cohort according to frequent mutations are reported in Supplementary Table S3.

**Fig. 2:**
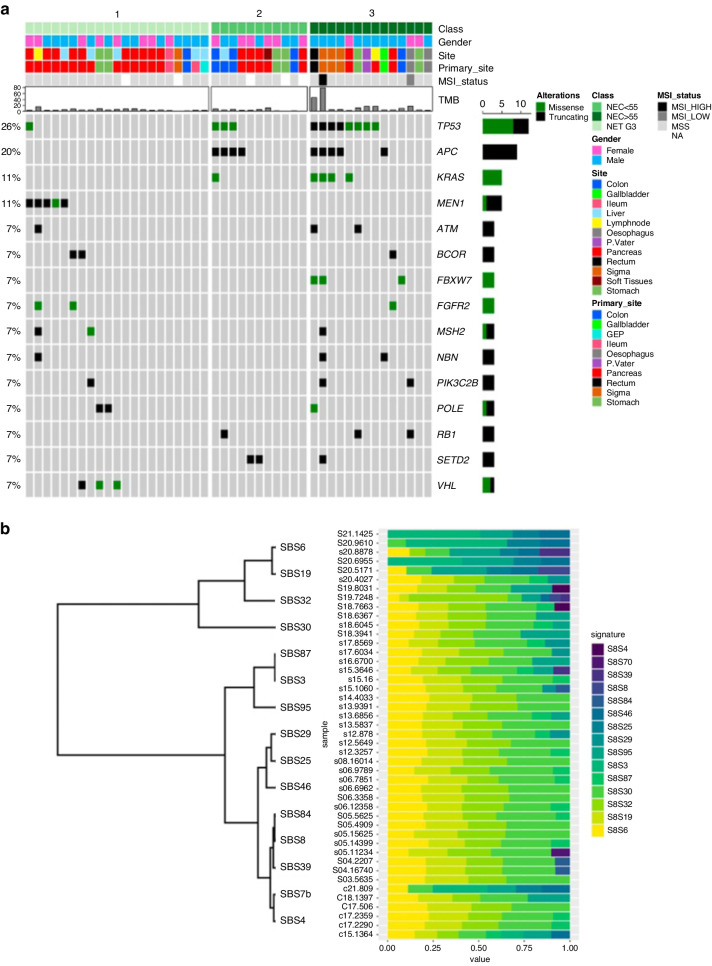
Genetic alterations and molecular signatures of high-grade GEP-NENs. **a** Oncoplot summarizing the genomic findings of 46 high-grade neuroendocrine neoplasms according to morphology and Ki-67. **b** Representation of COSMIC mutational signature analysis performed in 45 high-grade neuroendocrine neoplasms. NET neuroendocrine tumor, NEC neuroendocrine carcinoma.

Genomic profiling was performed in 46 of the 49 cases, because three samples yielded low DNA quality/quantity. Across the whole cohort the most frequently mutated genes were *TP53* (26.1%), *APC* (19.6%), *KRAS* (10.9%), and *MEN1* (10.9%); mutations in *ATM, BCOR, FBXW7, FGFR2, MSH2, NBN, PI3KC3B, POLE, RB1, SETD2*, and *VHL* genes were detected in 6.5% of patients (Fig. 2a). The analysis also identified amplifications in *MYC* and *FGFR1* in two samples (each 4.3%) and amplifications in *FGF5*, *MDM4*, *CCND3*, *FGF10*, *RICTOR*, *NRG1*, *MDM2* and *CDK4* genes in one sample (each 2.2%). Five fusion genes were detected (*SLC37A1*::*ERG*, *CNTN5*::*KMT2A*, *SEL1L*::*EGFR*, *FLT1*::*HUWE1*, *HFM1*::*ETV1*, and *BCL2*::*KCTD1)*, each in one sample (2.2%). Mutually exclusive and co-occurring analysis highlighted a co-occurrence between mutations in *APC* and *KRAS* (*p* = 0.003), *APC* and *TP53* (*p* = 0.0004), and *KRAS* and *TP53* (*p* = 0.0006). 22

Overall, the median TMB was 4.0 muts/Mb; 18.6% of tumors had a TMB ≥ 10 muts/Mb and 4.7% of tumors (two cases) had a TMB ≥ 20 muts/Mb. Of note, one of the two cases with a very high TMB (TMB ≥ 20 muts/Mb) was a POLE-mutated (S297F) rectal NEC ≥ 55% with a TMB of 46.4 muts/Mb.

MSI status was evaluated in 19 GEP-NET G3, 8 GEP-NECs <55%, and 14 GEP-NECs ≥55%. Only one MSI case was found in our cohort (2.4% of HG GEP-NENs and 4.5% of GEP-NECs), a colo-rectal (sigma) GEP-NECs ≥55% harboring an *MSH2* mutation (Q518Vfs*10) with a TMB of 79.3 muts/Mb.

#### GEP-NET G3

Among 21 GEP-NET G3, the most frequently mutated genes were *MEN1* (23.8%) and *VHL* (14.3%). All *MEN1* mutations and two out three *VHL* mutations were found in pancreatic NETs G3. Of note, both gastric NETs G3 were *POLE*-mutated. Amplifications in *MDM4* and *CCND3* and fusions in *SEL1L*::*EGFR*, and *HFM1*::*ETV1* were detected in one sample each (4.8%). Only one case (5.0%) had a TMB ≥ 10 muts/Mb.

#### GEP-NECs

Among 25 GEP-NECs, the most frequently mutated genes were: *TP53* (44.0%), *APC* (36.0%), *KRAS* (20.0%), *FBXW7* (12.0%), *RB1* (12.0%), and *SETD2* (12.0%). Amplifications in *MYC* and *FGFR1* were found in two cases each (8.0%) and fusions in *SLC37A1*::*ERG*, *CNTN5*::*KMT2A*, *FLT1*::*HUWE1*, and *BCL2*::*KCTD1* were detected in one sample each (4.0%). Overall, 30.4% of GEP-NECs had a TMB ≥ 10 muts/Mb.

In the subset of GEP-NECs <55% (*n* = 11), the most common genetic alterations were mutations in *APC* (36.4%), *TP53* (27.3%), and *SEDT2* (18.2%), and 11.1% of cases had TMB 10 muts/Mb.

Among GEP-NECs ≥55% (*n* = 14), the most common genetic alterations were mutations in *TP53* (57.1%), *APC* (35.7%), *KRAS* (28.6%), and *FBXW7* (21.4%), and 42.9% of cases had TMB ≥ 10 muts/Mb.

#### Genomic differences between GEP-NET G3, GEP-NECs < 55% and GEP-NECs ≥ 55%

GEP-NET G3 carried a higher frequency of genetic alterations in *MEN1* (23.8% *vs* 0% *vs* 0%; *p* = 0.02). On the other hand, GEP-NECs ≥55%, followed by GEP-NECs <55% showed a higher frequency of genetic alterations in *TP53* (57.1% *vs* 27.3% *vs* 4.8%; *p* = 0.001), *APC* (35.7% *vs* 36.4% *vs* 0.0%; *p* = 0.002) and *KRAS* (28.6% *vs* 9.1% *vs* 0.0%; *p* = 0.02). GEP-NECs ≥55%, followed by GEP-NECs <55%, were enriched in tumors with TMB ≥ 10 muts/Mb (42.9% vs 11.1% vs 5.0%; *p* = 0.01).

### Genomic profile of HG GEP-NENs according to the primary tumor site

Among HG non-pancreatic NENs (*n* = 26), the most frequent mutations were: *TP53* (34.6%), *APC* (30.8%), *KRAS* (15.4%), *FBXW7* (11.5%), *POLE* (11.5%), and RB1 (11.5%). Conversely, the most frequent mutations of HG pancreatic NENs (*n* = 22) were: *MEN1* (22.7%), *TP53* (13.6%), *FGFR2* (13.6%). Overall, the distribution of mutations in *TP53* (*p* = 0.007)*, APC* (*p* = 0.001), and *KRAS* (*p* = 0.03) was significantly different when comparing colorectal, gastroesophageal, small bowel-biliary tract and pancreatic primary site. Of note, HG colorectal NENs (*n* = 12) had the highest frequency of mutations in *TP53* (58.3%), *APC* (77.8%), and *KRAS* (80.0%).

### Integrative IHC and genomic analysis

Tumors with Rb1 loss had a higher frequency of cases with TMB ≥ 10 muts/Mb, in comparison with tumors with retained Rb1 (44.4% *vs* 11.8%, *p* = 0.045). Aberrant p53 staining was associated with mutations in *TP53* (*p* = 0.01) and *APC* (*p* = 0.02). Loss of Rb1 was associated with mutations in *TP53* (*p* = 0.005). Overall, p53 IHC yields a rate of false negatives of 9.1% and a rate of false positives of 58.3% when compared to *TP53* mutational analysis (Supplementary Table S4).

### Mutational signatures

Mutational signature analysis was obtained for 45 of 49 cases. The most frequent signatures were SBS30, SBS6, SBS32 and SBS19, which are associated with different defective DNA repair mechanisms. Of note, the SBS3 signature related to defective Homologous Recombination DNA repair deficiency (HRD)/*BRCA1/2* mutation was identified in 5 samples. SBS29 signature related to tobacco chewing was observed in other 5 samples. None of the *POLE*-mutated samples showed the SBS10a/b signature, associated with hypermutator tumors [two out of three *POLE* mutations were truncating and are not associated with a hypermutator phenotype [31]]. Similarly, the sample harboring a *BRCA2* mutation did not show the SBS3 signature, possibly because it was a variant of uncertain significance (Fig. 2b).

### Gene expression profiles of GEP-NET G3 *vs* GEP-NECs

**Fig. 3:**
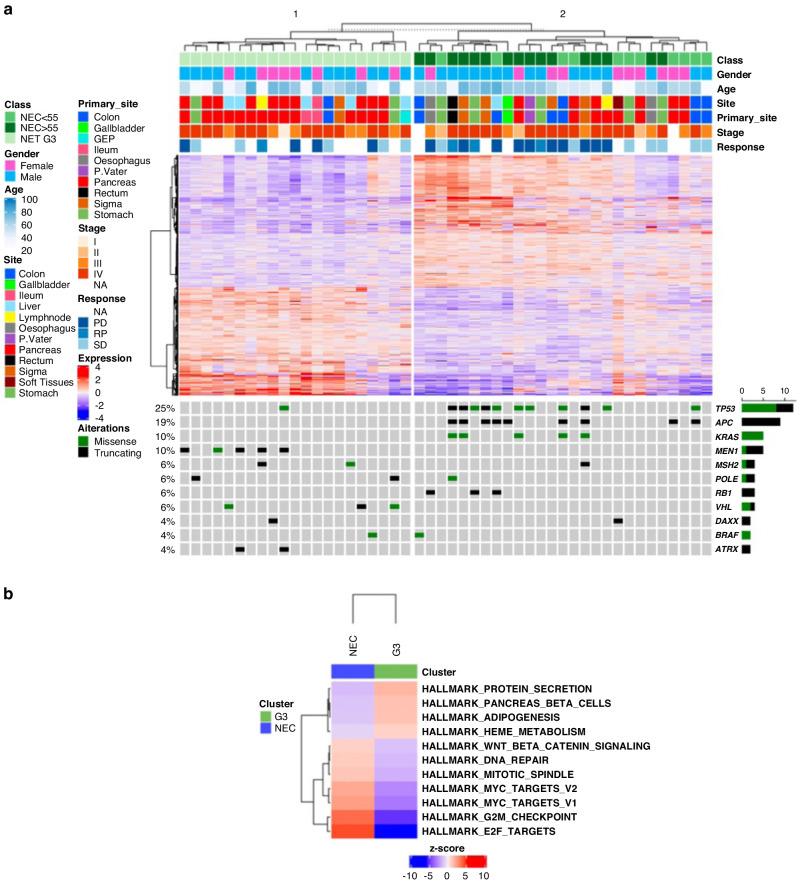
Transcriptomic profiling and pathway analysis of high-grade GEP-NENs. **a** Differential gene expression analysis of 48 high-grade neuroendocrine neoplasms, divided into neuroendocrine tumors (NET) G3 and neuroendocrine carcinomas (NECs). The expression values of the 1129 genes identified are arranged in rows. **b** Representation of up- and down-regulated pathways in NET G3 and NECs. Color legend: red and blue indicate high and low expression, respectively.

Due to preanalytical issues, RNA-seq was performed for 48 of the 49 RNA-suitable HG GEP-NENs. To identify the differentially expressed (DE) genes between GEP-NET G3, GEP-NEC < 55%, and GEP-NEC ≥ 55%, a supervised approach was used as follows. The cohort was divided into three groups (GEP-NET G3, GEP-NEC < 55%, GEP-NEC ≥ 55%), and each group was compared to the others (GEP-NET G3 *vs* GEP-NEC < 55% *vs* GEP-NEC ≥ 55%) in a pairwise comparison. As a result, we obtained 1574 DE genes for GEP-NET G3 *vs* GEP-NEC ≥ 55, 0 DE genes for GEP-NEC < 55% *vs* GEP-NEC ≥ 55% and 0 DE genes for GEP-NET G3 *vs* GEP-NEC < 55%. We subsequently merged the GEP-NEC < 55% and GEP-NEC ≥ 55 categories, as part of the same morphologic entity of GEP-NECs. Thus, we obtained two groups: GEP-NET G3 and GEP-NECs. A pairwise DE analysis was performed on these two categories (GEP-NET G3 and GEP-NEC) and a total of 1129 DE genes were identified using an adjusted *p*-value ≤ 0.05 as a significance threshold (Fig. 3a).

Gene set enrichment analysis (GSEA) performed on the two defined groups an association between adipogenesis, pancreatic beta cells and heme metabolism signatures with GEP-NET G3. This result was probably obtained due to the enrichment of pancreatic samples (*p* = 0.02). Conversely, the GEP-NECs group showed enrichment in Wnt-Beta catenin and Myc downstream signaling, DNA repair and mitogenic signaling (Fig. 3b).

### Gene expression profiles of pancreatic and non-pancreatic HG GEP-NENs

An expression-based molecular map was developed using UMAP method to understand the topological relationships between samples. A clear separation was observed between GEP-NET G3 and GEP-NEC ≥ 55% samples while GEP-NEC < 55% samples were differently distributed between two separated groups (Supplementary Fig. S1).

We subsequently divided the cohort according to the primary site in pancreatic (Pan) and non-pancreatic (Non-pan) samples to investigate the transcriptomic profile according to the tumor site. DE analysis was performed between 13 Pan-NET G3 and 8 Pan-NECs, but no DE genes were identified. When applying the same procedure on 8 Non-pan-NET G3 and 20 Non-pan-NECs, the analysis identified 450 DE genes (Supplementary Fig. S2A). *ASPM*, *CDCA7*, *CENPF* and *EPHB2* were upregulated in Non-pan-NET G3 while *CDHR3*, *FTCD*, *PTPRN* and *SERPINA1* in Non-pan-NEC (Supplementary Fig. S2B). Using GSEA, we observed that the Non-pan-NEC group was enriched in mitogenic processes, MYC and WNT signaling signatures. Conversely, adipogenesis was the only signature associated with Non-pan-NET G3 group (Supplementary Fig. S2C).

### Immune microenvironment

Next, we analyzed the gene expression profile related to the immune microenvironment. A low leukocyte infiltrate was globally observed in all samples. Moreover, enrichment of cancer-associated fibroblast (CAF), macrophages M2-like and endothelial cells was observed in GEP-NET G3 samples. Differently, the GEP-NEC group was enriched in CD4^+^ and CD8^+^ T cells. Among immune-related genes, *CTLA4* was up-regulated in GEP-NECs samples but the CTLA4 blockade signature was not overexpressed in GEP-NECs (Supplementary Fig. S3).

### Possible association between replication stress and disease progression after chemotherapy

Thirty of 48 patients underwent chemotherapy: 10 GEP-NET G3 and 20 GEP-NEC patients. Only patients with progressive disease (PD) and stable disease (SD) were included in the analysis, patients with partial responses were excluded. As a result, 9 GEP-NET G3 and 17 GEP-NECs samples were analyzed.

The GEP-NEC cohort included 17 patients, of which 4 were Pan and 13 Non-pan tumors. At follow-up 7 SD and 10 PD were reported. Supervised DE analysis between the PD and SD groups did not identify any DE genes, but showed a higher expression of *ARG2*, *E2F8*, *FAM222A* and *UHRF1* in the PD group, without reaching statistical significance (Supplementary Fig. S4A). Similarly, the GSEA showed an enrichment of several pathways implied in replication stress. Oncogene-selected expression analysis showed overexpression of *AURKA*, *CDC6*, *CDCA5*, *CCNE2*, *DEK*, *E2F1*, *HRAS* and *MYBL2* genes in PD group, while *CCND2*, *CCND3* and *STAT5A* genes were overexpressed in the SD group (Supplementary Fig. S4B). Of note, three of the PD samples with replicative stress signature showed the SBS3 signature in 2 cases and a truncating mutation in *BRCA2* gene in one sample. The SBS3 signature was observed only in 1 sample without replicative stress signature and SD performance.

The same analysis performed on GEP-NET G3 cohort did not identify any DE genes or enrichment in specific pathways.

### Survival analysis

**Fig. 4:**
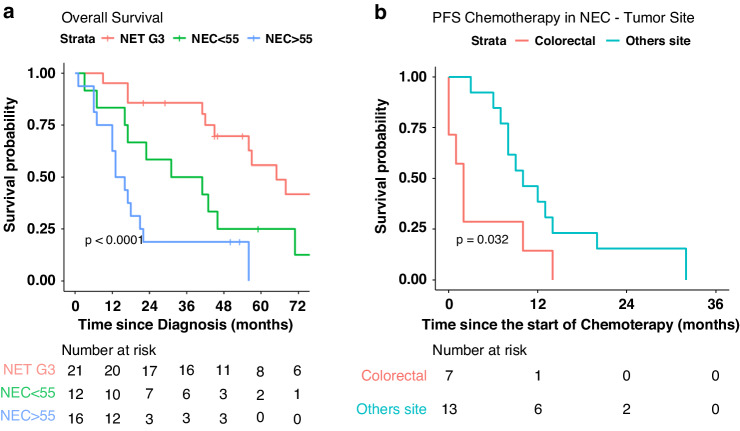
Survival outcomes in high-grade GEP-NENs. **a** Overall survival according to morphology and Ki-67. **b** Progression-free survival (PFS) after first-line chemotherapy in colorectal neuroendocrine carcinomas (NECs) *vs* NECs from other sites.

Survival analysis demonstrated the worst OS for GEP-NECs ≥55%, followed by GEP-NECs <55% (*p* < 0.0001) (Fig. 4a). p53 aberrant expression (*p* = 0.01), Rb1 absence of expression (*p* = 0.003), SSTR-2A absence of expression (*p* = 0.02), *TP53* (*p* = 0.0002)*, APC* (*p* < 0.0001), *KRAS* (*p* = 0.001) mutations and wild-type *MEN1* (*p* = 0.04) were also associated with worse OS at univariate analysis. At multivariate analysis, histologic type, and Rb1 absence of expression proved to be independent prognostic factors for OS (Table 2).

**Table 2:** Univariate and multivariate analysis of overall survival of patients with high-grade neuroendocrine neoplasms (49 patients)

Variable	Univariate HR (95% CI)	*p*-value	Adjusted for Site HR (95% CI)	*p*-value	Multivariate Model HR (95% CI)	*p*-value
Age (10-years Increase)	1.11 (0.92–1.34)	0.29	1.06 (0.87–1.30)	0.55		
Sex (Male *vs* Female)	0.88 (0.45–1.72)	0.71	0.58 (0.26–1.29)	0.18		
Stage (IV *vs* I-II-III)	1.19 (0.57–2.45)	0.64	1.14 (0.55–2.38)	0.72	1.08 (0.41–2.84)	0.87
Site						
Colorectal	1.00		1.00		1.00	
Ileum-duodenum-gallbladder	0.57 (0.18–1.85)	0.36	0.57 (0.18–1.85)	0.36	0.82 (0.24–2.81)	0.75
Gastroesophageal	0.85 (0.33–2.19)	0.74	0.85 (0.33–2.19)	0.74	0.47 (0.16–1.41)	0.18
Pancreas	0.52 (0.23–1.16)	0.11	0.52 (0.23–1.16)	0.11	0.57 (0.22–1.49)	0.26
Histology						
NET G3	1.00		1.00		1.00	
NEC < 55	2.88 (1.22–6.83)	**0.02**	2.91 (1.20–7.07)	**0.02**	3.39 (1.23–9.35)	**0.02**
NEC ≥ 55	5.98 (2.47–14.46)	**<0.0001**	6.00 (2.34–15.39)	**0.0001**	6.14 (2.12–17.75)	**0.0008**
Therapy						
None	1.00		1.00		1.00	
Others	1.39 (0.20–9.94)	0.74	2.30 (0.30–17.83)	0.42	3.19 (0.35–29.06)	0.30
Chemotherapy	1.87 (0.44–7.94)	0.40	2.26 (0.52–9.88)	0.27	1.67 (0.30–9.37)	0.56
SSA	0.75 (0.14–3.92)	0.73	0.88 (0.16–4.69)	0.88	0.98 (0.16–6.19)	0.99
IHC p53 (Absent or overexpressed *vs* heterogeneous)	2.57 (1.25–5.28)	**0.01**	3.07 (1.37–6.85)	**0.006**		
IHC Rb1 (Present *vs* Absent)	0.30 (0.13–0.65)	**0.003**	0.33 (0.14–0.76)	**0.009**	0.34 (0.12–0.95)	**0.04**
IHC SSTR2A (Present 2-3 *vs* Absent 0-1)	0.45 (0.22–0.90)	**0.02**	0.47 (0.23–0.99)	0.05		
TMB (>10 *vs* <10)	1.26 (0.54–2.95)	0.59	1.41 (0.59–3.38)	0.45		
*TP53* (Mut *vs* WT)	4.36 (1.98–9.60)	**0.0002**	4.34 (1.86–10.09)	**0.0007**		
*APC* (Mut *vs* WT)	6.69 (2.79–16.06)	**<0.0001**	7.40 (2.64–20.75)	**0.0001**		
*KRAS* (Mut *vs* WT)	5.72 (2.02–16.17)	**0.001**	5.20 (1.68–16.10)	**0.004**		
*MEN1* (Mut *vs* WT)	0.12 (0.02–0.90)	**0.04**	0.13 (0.02–1.02)	0.052		

The values in bold corresponding to the columns (*p*-values) indicate statistically significant values (i.e. < 0.05).

*NET* neuroendocrine tumor, *NEC* neuroendocrine carcinoma, *HR* hazard ratio, *CI* confidence interval, *IHC* immunohistochemistry, *p53* tumor suppressor p53, *Rb1* retinoblastoma-associated protein, *SSTR-2A* somatostatin receptor 2A, *TBM* tumor mutational burden, *TP53* tumor protein 53 gene, *WT* wild type, *APC* adenomatous polyposis coli gene, *KRAS* Kirsten rat sarcoma virus gene, *MEN1* multiple endocrine neoplasia type 1 gene.

When analyzing only the GEP-NET G3 subgroup, SSTR-2A expression proved to be associated with improved OS (*p* = 0.00036) (Supplementary Fig. S5). When analyzing only the GEP-NEC subgroup, at univariate analysis, stage IV (*p* = 0.045), Rb1 absence of expression (*p* = 0.03), *TP53* (*p* = 0.02) and *APC* mutations (*p* = 0.04) were associated with worse OS (Supplementary Figs. S6 and S7). At multivariate analysis, histologic type (GEP-NECs ≥55% vs GEP-NECs <55%) and primary site were independent prognostic factors (Supplementary Table S5).

Moreover, PFS after first-line chemotherapy was significantly worse for colorectal NECs (*p* = 0.032) than for NECs from other sites (Fig. 4b).

## Conclusions

HG GEP-NENs are a heterogeneous group of rare neoplasms with an unfavorable prognosis. Based on morphology and Ki-67, three groups with prognostic significance have been identified: GEP NET G3, GEP-NECs <55%, and GEP-NECs ≥55%. GEP-NECs <55% show lower response rates to PBC in comparison with GEP-NECs ≥55%. Moreover, there is increasing evidence that alkylating-based chemotherapy should be considered for GEP-NECs <55% [13]. However, the molecular landscape of these prognostic groups has never been analyzed before.

Overall, as previously reported by Venizelos et al. [16], *TP53*, *APC*, and *KRAS* were the most frequently mutated genes in our cohort of HG GEP-NENs. However, Venizelos et al. [16] reported a higher prevalence of mutations in these genes due to the enrichment of NECs in their cohort of HG GEP-NENs. In our series, we clearly showed that the genetic alterations found in GEP-NET G3 were different from those found in GEP-NECs <55% and GEP-NECs ≥55%, and none of the most frequently altered genes in GEP-NET G3 (*MEN1*, *VHL*, *ATRXX*), except for *POLE*, was found to be altered in GEP NECs. The genomic profile of our cohort of GEP-NET G3 reflects the enrichment in pancreatic NET G3 (66.7%). GEP-NET G3 lacking the genetic alterations that characterize NECs usually share genomic alterations (PIK3/mTOR pathway and in *DAXX*/*ATRX*, *PTEN*, *TSC2*) with GEP-NET G1/G2 of the same sites [32]. GEP-NECs <55% and GEP-NECs ≥55% shared some similarities in their genomic profile, both harboring high rates of mutations in *TP53* and *APC*. However, GEP-NECs ≥55% had a TMB in comparison with GEP-NECs <55%.

Our findings from differential gene expression analysis between the three prognostic groups and afterward between GEP-NET G3 and GEP-NECs further corroborate the hypothesis that, while GEP-NETs G3 represent a separate morphologic and molecular entity, GEP-NECs <55% and GEP-NECs ≥55% may be part of the same molecular spectrum. In fact, a total of 1129 DE genes implicated in Wnt-Beta catenin and Myc signaling, DNA-repair and mitogenic signaling were identified between GEP-NET G3 and GEP-NECs. *APC* mutations, which are frequent in GEP-NECs, drive Wnt-Beta catenin pathway activation.

In contrast, GEP-NET G3 showed a strong association with the pancreatic beta cell signature, due to the overrepresentation of pancreatic samples. Gene expression profiling reveals the dysregulated pathways related to the pathogenesis of HG GEP-NENs. The results of our differential gene expression analysis suggest that GEP-NECs <55% and GEP-NECs ≥55% share common pathogenetic pathways while having different clinical outcomes, response to chemotherapy, and genomic alterations. Conversely, GEP-NETs G3 have similar pathogenetic mechanisms with GEP-NETs G1/G2, several of which are also related to familial syndromes [32].

The biology and clinical outcome of GEP-NENs are not exclusively dependent on morphology and proliferation rate, but also on the site of origin [5, 16]. Gastrointestinal and pancreatic NENs have proved to have differences in survival outcomes and clinical course [33]. Thus, different therapeutic approaches and surveillance strategies are adopted [34]. Further validating these findings, Puccini et al. [15] showed that gastrointestinal and pancreatic NENs harbor a different molecular profile. Interestingly, it has been found that some molecular features of colorectal and pancreatic NECs have similarities to their adenocarcinoma counterparts of the primary site [35, 36]. In our cohort, we found genomic differences not only between HG gastrointestinal and pancreatic NENs, but also related to the primitive site (i.e., gastroesophageal, small bowel and gallbladder, colorectal, and pancreas). Notably, in our analysis, HG colorectal NENs and colorectal NECs harbored a drastically higher proportion of *APC* mutations, than other groups. Similar trends were observed also for *KRAS* and *TP53*. Only one *BRAF* mutation was found in a colorectal NEC ≥ 55%. On the contrary, Venizelos et al. [16] reported that 49% of colonic and 8% of rectal NECs harbored *a BRAF* mutation. Chen et al. [37] found that *APC* and *KRAS*, which are frequently mutated in colorectal adenocarcinoma, were also commonly altered in colorectal NECs, unlike GEP-NETs and lung NECs [38, 39], suggesting that cell-cycle regulation, Wnt signaling, and MAPK and PI3K signaling are frequently aberrant in colorectal NECs. Furthermore, our analysis also highlights that the clinical outcome of patients with colorectal NECs treated with first-line Cisplatin plus Etoposide chemotherapy is worse than that of other gastrointestinal NECs. The similarities in terms of genomic and targetable alterations between colorectal NECs and colorectal adenocarcinomas further support the use of FOLFOX (folinic acid, fluorouracil, oxaliplatin) and FOLFIRI (folinic acid, fluorouracil, irinotecan) as second-line chemotherapy treatments in colorectal NEC, as recommended international guidelines [40, 41]. To further explore the differences between HG pancreatic and gastrointestinal NENs and to overcome the bias of enrichment of pancreatic NET G3 samples, we divided our cohort into Pan tumors and Non-Pan tumors. In our cohort, we did not identify any DE gene between Pan NET G3 and Pan NECs. Conversely, Yachida et al. [21] found Pan-NET G3 and Pan-NECs to have distinct genomic and transcriptomic profiles. The same analysis performed in Non-pan tumors identified 450 DE genes involved in mitogenic processes or in MYC and WNT signaling, confirming the results previously obtained from the entire cohort and suggesting that Non-Pan NET G3 and Non-Pan NECs may not be etiologically related.

In the past decade, immune checkpoint inhibitors (ICIs) have revolutionized cancer therapy. Several agents targeting programmed cell death protein 1 (PD-1), its ligand (PD-L1), and cytotoxic T-lymphocyte-associate protein 4 (CTLA4) have been investigated in GEP-NENs. However, the outcomes of these clinical trials have not been satisfactory [42–44]. Milione et al. [45] reported a clear difference in the immune-related profile of LG GEP-NENs *vs* HG GEP-NENs. According to the same study, at least a subset of HG GEP-NENs has microenvironment features consistent with spontaneous activation of adaptive immunity, suggesting potential for responsiveness to ICs [45]. It is crucial to gain insight into existing and emerging immune biomarkers in HG GEP-NENs, including MSI status, high-TMB, PD-L1 expression and microenvironment features, to improve patient stratification. Across our entire cohort, only one colorectal NECs ≥55% was MSI (2.4% of HG GEP-NENs and 4.5% of GEP-NECs). Venizelos et al. [16] also reported relatively low rates of MSI in their cohort: 5.4% among GEP-NECs and 3.4% among GEP-NET G3. Furthermore, TMB ≥ 10 mut/Mb, was more frequently observed in GEP-NECs, especially in GEP-NECs ≥55%. Despite previous studies reporting higher PD-L1 expression in HG GEP-NENs [15], in our cohort PD-L1 expression was absent in all the cases. The immune microenvironment landscape was found to be poor for both GEP NET G3 and GEP NECs, with a slight enrichment of CD4 and CD8 T cells in GEP-NECs and of cancer-associated fibroblasts and macrophages (M2-like) in GEP-NET G3. Of note, the *CTLA4* gene was differentially expressed with enrichment in GEP-NECs, but no immunotherapy-related signature was associated with either group. Overall, GEP-NECs appear to be more immunogenic than GEP-NET G3. However, our results pinpoint the molecular rationale behind the modest activity of ICIs in HG GEP-NEN patients.

As reported by Alexandrov et al. [46], the signatures identified in our cases are compatible with those present in the 81 Panc-Endocrine cases of their study. In particular, the frequent presence of the SBS6 and SBS30 signatures in the absence of causative mutations is compatible with a genome subject to various genomic alterations as described in Scarpa et al. [47]. Similarly, the presence of the SBS3 signature linked to HRD was observed in five samples; however, no mutation in *BRCA1/2* or other HRD-related genes was observed. Conversely, the only case affected by a truncating mutation of *BRCA2* did not have this signature. A study by Dreyer et al. [48] on pancreatic adenocarcinomas highlighted the presence of a HRD signature in the absence of mutations in the genes belonging to the same pathway. The characterization of NEC cases with SD following chemotherapy treatment highlighted how replicative stress is a mechanism to be taken into consideration in the stratification of these patients. Our results suggest that replicative stress signature and SBS3 signature in the absence of *BRCA1/2* genetic alterations may be associated with PD after chemotherapy. Similarly, the overexpression of genes such as *STAT5A*, *CCND2* and *CCND3* could be a surrogate marker to select these responsive patients. Our findings are of clinical and therapeutic interest because replication stress is being targeted by inhibiting kinases that coordinate the DNA damage response with cell cycle control, including ATR, CHK1, WEE1, and MYT1 checkpoint kinases. At present, several ATR, CHK1, WEE1, and MYT1 inhibitors are undergoing clinical evaluation as monotherapy or combinatorial regimens [49].

The ESMO 2020 GEP-NEN guidelines [4] recommend using *RB1* mutations to discriminate between GEP-NET G3 and GEP-NECs. Our findings suggest that testing for Rb1 loss by means of IHC is a more reliable approach than mutational testing. However, an ancillary IHC panel comprising Rb1, p53, and SSTR-2A would be the most accurate in distinguishing between GEP-NET G3 and GEP-NECs in routine practice. Testing for Rb1 loss would provide prognostic information since it proved to be an independent marker of poor prognosis in our cohort of GEP-NECs. Of note, the use of p53 IHC to identify *TP53* mutations should not recommended due to the high rate of false positives.

A limitation of the study is represented by the small number of cases especially in the cohort of patients undergoing chemotherapy and by the enrichment of pancreatic cases in the GEP-NET G3 group. To conclude, the heterogeneity of HG GEP-NENs in terms of morphology and site of origin is reflected at the molecular level. GEP-NET G3 and GEP-NECs exhibit clear genomic and transcriptomic differences, while GEP-NECs <55% and GEP-NECs ≥55% may be part of the same molecular spectrum. We also provided molecular findings with prognostic and predictive value regarding immune biomarkers and microenvironment features, response to chemotherapy and survival.

### Supplementary information

Supplementary Information

## Data Availability

The data that support the findings of this study are available on request from the corresponding author.
