# Supplementary material for: Comprehensive genomic and transcriptomic characterization of high-grade gastro-entero-pancreatic neoplasms
Source: Br J Cancer. 2024 May 10;131(1):159–70. doi: 10.1038/s41416-024-02705-8 (PMC11231306; doi:10.1038/s41416-024-02705-8)
Supplement: Supplementary file 1 — Supplementary Information [file 41416_2024_2705_MOESM1_ESM.docx]

**SUPPLEMENTARY INFORMATION**

**Patients and samples**

Patients’ charts and tumor morphology were carefully revised, first by a panel of three expert pathologists (MM, GS, and VA) using a multi-headed microscope. During panel meetings, the original diagnosis was reviewed, and further workup was carried out whenever panelists disagreed, or quantitative evaluations approached cut-off values. For qualitative parameters, a majority decision was adopted, while for quantitative evaluations the mean of values obtained by the individual panelists was taken as final. Neuroendocrine identification was based on parallel investigation of at least two consecutive sections from representative blocks, stained with hematoxylin-eosin and then with immunohistochemistry for synaptophysin and chromogranin-A. Ki-67 proliferative rate (or other histochemical parameters investigated) was assessed on further consecutive sections.

The following analyses were performed. About neuroendocrine neoplasm (NEN) morphology, well-differentiated (WD) NENs and poorly differentiated (PD) NENs were distinguished according to different criteria. In details, WD NENs defined as neuroendocrine tumors were characterized by microlobular, microacinar, or thinly trabecular structures formed by relatively monomorphic, regularly arranged cells with fine chromatin and inconspicuous nucleoli. PD NENs defined as neuroendocrine carcinomas showed poorly formed, large trabeculae and solid nests or diffuse sheets of cells frequently showing multiple necrotic foci or large and confluent areas of “geographic chart” necrosis and brisk mitotic activity.

**Supplementary Table S1.** Antibody sources and dilutions.

| **Antigens** | **Dilution** | **Code Number** | **Clone** | **Source** |
| --- | --- | --- | --- | --- |
| Ki-67 (M) | 1/400 | M7240 | Mib-1 | Dako, Glostrup, Denmark |
| Synaptophysin (M) | 1/200 | M7315 | Dak-Synap | Dako |
| Chromogranin-A (M) | 1/100 | M0869 | Dak-A3 | Dako |
| p53 (M) | 1/50 | M7001 | DO-7 | Dako |
| SSTR-2A (M) | 1/800 | AB134152 | UMB1 | Abcam |
| Rb1 PMG (M) | 1/200 | 554136 | G3-245 | BD/Pharmingen San Diego,CA, USA |
| PD-L1 (M) | Prediluted | SK006 | 22C3 | Agilent-Dako |

Abbreviation: M, monoclonal antibody; p53, tumor suppressor p53; SSTR-2A, somatostatin receptor 2A; Rb1, retinoblastoma-associated protein; PD-L1, programmed death-ligand 1.

**Supplementary Table S2.** Characteristics of patients with high-grade neuroendocrine neoplasms according to WHO Class.

|  | | | | |
| --- | --- | --- | --- | --- |
|  | **All patients** | **NET G3** | **NEC** | **p-value*** |
|  |  |  |  |  |
| **Total** | 49 (100) | 21 (100) | 28 (100) |  |
| **Gender** |  |  |  |  |
| Female | 18 (36.7) | 8 (38.1) | 10 (35.7) |  |
| Male | 31 (63.3) | 13 (61.9) | 18 (64.3) | 1.0 |
| **Age** |  |  |  |  |
| Mendian [range] | 61 [21-87] | 58 [21-80] | 61 [26-87] | 0.6 |
| **Stage** |  |  |  |  |
| I | 1 (2.1) | 1 (4.8) | 0 (0.0) |  |
| II | 3 (6.4) | 0 (0.0) | 3 (11.5) |  |
| III | 13 (27.7) | 6 (28.6) | 7 (26.9) |  |
| IV | 30 (63.8) | 14 (66.7) | 16 (61.5) | 0.3 |
| **Site primitive** |  |  |  |  |
| Colorectal | 12 (24.5) | 2 (9.5) | 10 (35.7) |  |
| Gastroesophageal | 9 (18.4) | 2 (9.5) | 7 (25.0) |  |
| Ileum-duodenum-gallbladder | 6 (12.2) | 3 (14.3) | 3 (10.7) |  |
| Pancreas | 22 (44.9) | 14 (66.7) | 8 (28.6) | **0.03** |
| **Site Primitive** |  |  |  |  |
| Pancreas | 22 (44.9) | 14 (66.7) | 8 (28.6) |  |
| Other | 27 (55.1) | 7 (33.3) | 20 (71.4) | **0.01** |
| **Therapy** |  |  |  |  |
| None | 5 (10.6) | 3 (14.3) | 2 (7.7) |  |
| Chemotherapy | 30 (63.8) | 10 (47.6) | 20 (77.0) |  |
| Others | 3 (6.4) | 2 (9.5) | 1 (3.8) |  |
| SSA | 9 (19.1) | 6 (28.6) | 3 (11.5) | 0.2 |
| ***Molecular Features*** |  |  |  |  |
| **TMB** |  |  |  |  |
| Mendian [range] | 4.0 [0.8-79.3] | 3.9 [0.8-14.9] | 5.5 [0.8-79.3] | 0.2 |
| **TMB** | |  |  |  |
| <10 | 35 (81.4) | 19 (95.0) | 16 (69.6) |  |
| >10 | 8 (18.6) | 1 (5.0) | 7 (30.4) | 0.05036 |
| ***TP53*** |  |  |  |  |
| WT | 34 (73.9) | 20 (95.2) | 14 (56.0) |  |
| Mutated | 12 (26.1) | 1 (4.8) | 11 (44.0) | **0.003** |
| ***APC*** |  |  |  |  |
| WT | 37 (80.4) | 21 (100.0) | 16 (64.0) |  |
| Mutated | 9 (19.6) | 0 (0.0) | 9 (36.0) | **0.002** |
| ***KRAS*** |  |  |  |  |
| WT | 41 (89.1) | 21 (100.0) | 20 (80.0) |  |
| Mutated | 5 (10.9) | 0 (0.0) | 5 (20.0) | 0.0536 |
| ***MEN1*** |  |  |  |  |
| WT | 41 (89.1) | 16 (76.2) | 25 (100) |  |
| Mutated | 5 (10.9) | 5 (23.8) | 0 (0.0) | **0.01** |
| ***Immunohistochemical Markers*** |  |  |  |  |
| **p53** |  |  |  |  |
| Absent | 19 (38.8) | 8 (38.1) | 11 (39.3) |  |
| Heterogeneus | 22 (44.9) | 12 (57.1) | 10 (35.7) |  |
| Overexpressed | 8 (16.3) | 1 (4.8) | 7 (25.0) | 0.1 |
| **Rb1** |  |  |  |  |
| Absent | 10 (20.4) | 1 (4.8) | 9 (32.1) |  |
| Present | 39 (79.6) | 20 (95.2) | 19 (67.9) | **0.03** |
| **SSTR-2A** |  |  |  |  |
| Absent (0-1) | 23 (46.9) | 6 (28.6) | 17 (60.7) |  |
| Present (2-3) | 26 (53.1) | 15 (71.4) | 11 (39.3) | **0.04** |
| Note: * p-value based on the Fisher’s exact for categorical variables or Wilcoxon-Mann-Whitney test for continuos variables. Abbreviation: NET, neuroendocrine tumor; NEC, neuroendocrine carcinoma; SSA, somatostatin analogues; TBM, tumor mutational burden; *TP53*, tumor protein 53 gene; WT, wild type; *APC*, adenomatous polyposis coli gene; *KRAS*, Kirsten rat sarcoma virus gene; *MEN1*, multiple endocrine neoplasia type 1 gene; p53, tumor suppressor p53; Rb1, retinoblastoma-associated protein; SSTR-2A, somatostatin receptor 2A. | | | | |

| **Supplementary Table S3.** Characteristics of patients with high-grade neuroendocrine neoplasms according to frequent mutations. | | | | | | | | | | | | | | | | | |
| --- | --- | --- | --- | --- | --- | --- | --- | --- | --- | --- | --- | --- | --- | --- | --- | --- | --- |
|  | **All patients** | ***TP53*  WT** | ***TP53*  MUT** | **p-value*** | **p-value#** | ***APC*  WT** | ***APC*  MUT** | **p-value*** | **p-value#** | ***KRAS* WT** | ***KRAS* MUT** | **p-value*** | **p-value#** | ***MEN1* WT** | ***MEN1* MUT** | **p-value*** | **p-value§** |
|  |  |  |  |  |  |  |  |  |  |  |  |  |  |  |  |  |  |
| **Total** | 49 (100) | 34 (100) | 12 (100) |  |  | 37 (100) | 9 (100) |  |  | 41 (100) | 5 (100) |  |  | 41 (100) | 5 (100) |  |  |
| **Age** |  |  |  |  |  |  |  |  |  |  |  |  |  |  |  |  |  |
| Median [range] | 61 [21-87] | 58.5 [21-80] | 67.5 [39-79) | **0.03** | **0.04** | 60 [21-80] | 67 [39-79] | 0.22 | 0.28 | 61 [21-80] | 48 [39-76] | 0.83 | 0.56 | 61 [21-80] | 58 [28-74] | 0.94 | 1.00 |
| **Gender** |  |  |  |  |  |  |  |  |  |  |  |  |  |  |  |  |  |
| Female | 18 (36.7) | 14 (41.2) | 3 (25.0) |  |  | 15 (40.5) | 2 (22.2) |  |  | 15 (36.6) | 2 (40.0) |  |  | 15 (36.6) | 2 (40.0) |  |  |
| Male | 31 (63.3) | 20 (58.8) | 9 (75.0) | 0.49 | 0.20 | 22 (59.5) | 7 (77.8) | 0.45 | 0.40 | 26 (63.4) | 3 (60.0) | 1.00 | 1.00 | 26 (63.4) | 3 (60.0) | 1.00 | 1.00 |
| **Histology** |  |  |  |  |  |  |  |  |  |  |  |  |  |  |  |  |  |
| NET G3 | 21 (42.9) | 20 (58.8) | 1 (8.3) |  |  | 21 (56.8) | 0 (0.0) |  |  | 21 (51.2) | 0 (0.0) |  |  | 16 (39.0) | 5 (100) |  |  |
| NEC <55 | 12 (24.5) | 8 (23.5) | 3 (25.0) |  |  | 7 (18.9) | 4 (44.4) |  |  | 10 (24.4) | 1 (20.0) |  |  | 11 (26.8) | 0 (0.0) |  |  |
| NEC ≥55 | 16 (32.7) | 6 (17.7) | 8 (66.7) | **0.001** | 0.22 | 9 (24.3) | 5 (55.6) | **0.002** | 1.00 | 10 (24.4) | 4 (80.0) | **0.01** | 0.34 | 14 (34.1) | 0 (0.0) | 0.06 | - |
| **Stage** |  |  |  |  |  |  |  |  |  |  |  |  |  |  |  |  |  |
| I-II-III | 17 (36.2) | 13 (40.6) | 3 (25.0) |  |  | 14 (38.9) | 2 (25.0) |  |  | 14 (35.9) | 2 (40.0) |  |  | 15 (38.5) | 1 (20.0) |  |  |
| IV | 30 (63.8) | 19 (59.4) | 9 (75.0) | 0.49 | 0.08 | 22 (61.1) | 6 (75.0) | 0.68 | 0.40 | 25 (64.1) | 3 (60.0) | 1.00 | 1.00 | 24 (61.5) | 4 (80.0) | 0.64 | 0.62 |
| **Site** |  |  |  |  |  |  |  |  |  |  |  |  |  |  |  |  |  |
| Colorectal | 12 (24.5) | 3 (8.8) | 7 (58.3) |  |  | 3 (8.1) | 7 (77.8) |  |  | 6 (14.6) | 4 (80.0) |  |  | 10 (24.4) | 0 (0.0) |  |  |
| Gastroesophageal | 9 (18.4) | 7 (20.6) | 1 (8.3) |  |  | 8 (21.6) | 0 (0.0) |  |  | 8 (19.5) | 0 (0.0) |  |  | 8 (19.5) | 0 (0.0) |  |  |
| Ileum-duodenum-gallbladder | 6 (12.2) | 5 (14.7) | 1 (8.3) |  |  | 5 (13.5) | 1 (11.1) |  |  | 6 (14.6) | 0 (0.0) |  |  | 6 (14.6) | 0 (0.0) |  |  |
| Pancreas | 22 (44.9) | 19 (55.9) | 3 (25.0) | **0.007** | **0.02** | 21 (56.8) | 1 (11.1) | **0.0001** | **0.0008** | 21 (51.2) | 1 (20.0) | **0.03** | 0.13 | 17 (41.5) | 5 (100) | 0.15 | 0.64 |
| **TMB** | |  |  |  |  |  |  |  |  |  |  |  |  |  |  |  |  |
| <10 | 35 (81.4) | 28 (90.3) | 7 (58.3) |  |  | 28 (82.4) | 7 (77.8) |  |  | 32 (84.2) | 3 (60.0) |  |  | 31 (81.6) | 4 (80.0) |  |  |
| >10 | 8 (18.6) | 3 (9.7) | 5 (41.7) | **0.03** | 0.20 | 6 (17.6) | 2 (22.2) | 1.00 | 0.65 | 6 (15.8) | 2 (40.0) | 0.23 | 0.62 | 7 (18.4) | 1 (20.0) | 1.00 | 0.25 |
| ***TP53*** |  |  |  |  |  |  |  |  |  |  |  |  |  |  |  |  |  |
| WT | 34 (73.9) | - | - |  |  | 32 (86.5) | 2 (22.2) |  |  | 34 (82.9) | 0 (0.0) |  |  | 30 (73.2) | 4 (80.0) |  |  |
| Mutated | 12 (26.1) | - | - |  |  | 5 (13.5) | 7 (77.8) | **0.0004** | **0.02** | 7 (17.1) | 5 (100.0) | **0.0006** | **0.009** | 11 (26.8) | 1 (20.0) | 1.00 | 0.24 |
| ***APC*** |  |  |  |  |  |  |  |  |  |  |  |  |  |  |  |  |  |
| WT | 37 (80.4) | - | - |  |  | - | - |  |  | 36 (87.8) | 1 (20.0) |  |  | 32 (78.0) | 5 (100) |  |  |
| Mutated | 9 (19.6) | - | - |  |  | - | - |  |  | 5 (12.2) | 4 (80.0) | **0.003** | **0.04** | 9 (22.0) | 0 (0.0) | 0.57 | - |
| ***KRAS*** |  |  |  |  |  |  |  |  |  |  |  |  |  |  |  |  |  |
| WT | 41 (89.1) | - | - |  |  | - | - |  |  | - | - |  |  | 36 (87.8) | 5 (100) |  |  |
| Mutated | 5 (10.9) | - | - |  |  | - | - |  |  | - | - |  |  | 5 (12.2) | 0 (0.0) | 1.00 | - |

Note: * p-value based on the Fisher’s exact for categorical variables and the Wilcoxon test for continuous variables; # p-value evaluated in NEC only; § p-value evaluated in NET G3 only. Abbreviation: NET, neuroendocrine tumor; NEC, neuroendocrine carcinoma; TBM, tumor mutational burden; *TP53*, tumor protein 53 gene; WT, wild type; MUT, mutated; *APC*, adenomatous polyposis coli gene; *KRAS*, Kirsten rat sarcoma virus gene; *MEN1*, multiple endocrine neoplasia type 1 gene.

| **Supplementary Table S4**. Characteristics of patients with high-grade neuroendocrine neoplasms according to immunohistochemical profile. | | | | | | | | | | | | | |
| --- | --- | --- | --- | --- | --- | --- | --- | --- | --- | --- | --- | --- | --- |
|  | **All patients** | **p53  Heterogeneous** | **p53 absent or overexpressed** | **p-value*** | **p-value#** | **Rb1 Absent** | **Rb1 Present** | **p-value*** | **p-value#** | **SSTR-2A Absent (0-1)** | **SSTR-2A Present (2-3)** | **p-value*** | **p-value#** |
|  |  |  |  |  |  |  |  |  |  |  |  |  |  |
| **Total** | 49 (100) | 22 (100) | 27 (100) |  |  | 10 (100) | 39 (100) |  |  | 23 (100) | 26 (100) |  |  |
| **Age** |  |  |  |  |  |  |  |  |  |  |  |  |  |
| Median [range] | 61 [21-87] | 61 [28-80] | 61 [21-87] | 0.35 | 0.32 | 67.5 [21-79] | 60 [25-87] | 0.18 | **0.03** | 61 [21-87] | 59 [26-80] | 0.61 | 0.49 |
| **Gender** |  |  |  |  |  |  |  |  |  |  |  |  |  |
| Female | 18 (36.7) | 10 (45.5) | 8 (29.6) |  |  | 1 (10.0) | 17 (43.6) |  |  | 8 (34.8) | 10 (38.5) |  |  |
| Male | 31 (63.3) | 12 (54.5) | 19 (70.4) | 0.37 | 0.41 | 9 (90.0) | 22 (56.4) | 0.07 | 0.10 | 15 (65.2) | 16 (61.5) | 1.00 | 0.44 |
| **Histology** |  |  |  |  |  |  |  |  |  |  |  |  |  |
| NET G3 | 21 (42.9) | 12 (54.5) | 9 (33.3) |  |  | 1 (10.0) | 20 (51.3) |  |  | 6 (26.1) | 15 (57.7) |  |  |
| NEC <55 | 12 (24.5) | 5 (22.7) | 7 (25.9) |  |  | 1 (10.0) | 11 (28.2) |  |  | 7 (30.4) | 5 (19.2) |  |  |
| NEC ≥55 | 16 (32.7) | 5 (22.7) | 11 (40.7) | 0.31 | 0.70 | 8 (80.0) | 8 (20.5) | **0.002** | **0.04** | 10 (43.5) | 6 (23.1) | 0.10 | 1.00 |
| **Stage** |  |  |  |  |  |  |  |  |  |  |  |  |  |
| I-II-III | 17 (36.2) | 9 (40.9) | 8 (32.0) |  |  | 1 (11.1) | 16 (42.1) |  |  | 10 (47.6) | 7 (26.9) |  |  |
| IV | 30 (63.8) | 13 (59.1) | 17 (68.0) | 0.56 | 0.11 | 8 (88.9) | 22 (57.9) | 0.13 | 0.10 | 11 (52.4) | 19 (73.1) | 0.22 | 0.43 |
| **Site** |  |  |  |  |  |  |  |  |  |  |  |  |  |
| Colorectal | 12 (24.5) | 2 (9.1) | 10 (37.0) |  |  | 5 (50.0) | 7 (17.9) |  |  | 7 (30.4) | 5 (19.2) |  |  |
| Gastroesophageal | 9 (18.4) | 6 (27.3) | 3 (11.1) |  |  | 3 (30.0) | 6 (15.4) |  |  | 5 (21.7) | 4 (15.4) |  |  |
| Ileum-duodenum-gallbladder | 6 (12.2) | 3 (13.6) | 3 (11.1) |  |  | 1 (10.0) | 5 (12.8) |  |  | 2 (8.7) | 4 (15.4) |  |  |
| Pancreas | 22 (44.9) | 11 (50.0) | 11 (40.7) | 0.11 | **0.004** | 1 (10.0) | 21 (53.8) | **0.03** | 0.10 | 9 (39.1) | 13 (50.0) | 0.66 | 0.86 |
| **TMB** | |  |  |  |  |  |  |  |  |  |  |  |  |
| <10 | 35 (81.4) | 17 (85.0) | 18 (78.3) |  |  | 5 (55.6) | 30 (88.2) |  |  | 15 (75.0) | 20 (87.0) |  |  |
| >10 | 8 (18.6) | 3 (15.0) | 5 (21.7) | 0.70 | 0.66 | 4 (44.4) | 4 (11.8) | **0.045** | 0.18 | 5 (25.0) | 3 (13.0) | 0.44 | 0.66 |
| ***TP53*** |  |  |  |  |  |  |  |  |  |  |  |  |  |
| WT | 34 (73.9) | 20 (90.9) | 14 (58.3) |  |  | 3 (33.3) | 31 (83.8) |  |  | 15 (68.2) | 19 (79.2) |  |  |
| Mutated | 12 (26.1) | 2 (9.1) | 10 (41.7) | **0.01** | 0.10 | 6 (66.7) | 6 (16.2) | **0.005** | 0.08 | 7 (31.8) | 5 (20.8) | 0.51 | 1.00 |
| ***APC*** |  |  |  |  |  |  |  |  |  |  |  |  |  |
| WT | 37 (80.4) | 21 (95.5) | 16 (66.7) |  |  | 5 (55.5) | 32 (86.5) |  |  | 15 (68.2) | 22 (91.7) |  |  |
| Mutated | 9 (19.6) | 1 (4.5) | 8 (33.3) | **0.02** | **0.04** | 4 (44.5) | 5 (13.5) | 0.06 | 0.39 | 7 (31.8) | 2 (8.3) | 0.07 | 0.40 |
| ***KRAS*** |  |  |  |  |  |  |  |  |  |  |  |  |  |
| WT | 41 (89.1) | 21 (95.5) | 20 (83.3) |  |  | 7 (77.8) | 34 (91.9) |  |  | 19 (86.4) | 22 (91.7) |  |  |
| Mutated | 5 (10.9) | 1 (4.5) | 4 (16.7) | 0.35 | 0.61 | 2 (22.2) | 3 (8.1) | 0.25 | 1.00 | 3 (13.6) | 2 (8.3) | 0.66 | 1.00 |
| ***MEN1*** |  |  |  |  |  |  |  |  |  |  |  |  |  |
| WT | 41 (89.1) | 19 (86.4) | 22 (91.7) |  |  | 9 (100) | 32 (86.5) |  |  | 21 (95.5) | 20 (83.3) |  |  |
| Mutated | 5 (10.9) | 3 (13.6) | 2 (8.3) | 0.66 | 1.00 | 0 (0.0) | 5 (13.5) | 0.57 | 1.00 | 1 (4.5) | 4 (16.7) | 0.35 | 1.00 |

Note: * p-value based on the Fisher’s exact for categorical variables and the Wilcoxon test for continuous variables; # p-value evaluated in NEC only; § p-value evaluated in NET G3 only. Abbreviation: NET, neuroendocrine tumor; NEC, neuroendocrine carcinoma; TBM, tumor mutational burden; *TP53*, tumor protein 53 gene; WT, wild type; *APC*, adenomatous polyposis coli gene; *KRAS*, Kirsten rat sarcoma virus gene; *MEN1*, multiple endocrine neoplasia type 1 gene; p53, tumor suppressor p53; Rb1, retinoblastoma-associated protein; SSTR-2A, somatostatin receptor 2A.

**Supplementary Table S5.** Univariate and multivariate analysis of overall survival of patients with neuroendocrine carcinoma (28 patients).

| **Variable** | **Univariate  HR (95% CI)** | **p-value** | | **Adjusted for Site HR (95% CI)** | **p-value** | **Multivariate Model HR (95% CI)** | **p-value** |
| --- | --- | --- | --- | --- | --- | --- | --- |
| **Age** (10-years Increase) | 1.00 (0.79-1.29) | | 0.96 | 1.00 (0.77-1.31) | 0.99 |  |  |
| **Sex** (Male *vs* Female) | 0.78 (0.34-1.76) | | 0.55 | 0.35 (0.10-1.21) | 0.10 |  |  |
| **Stage** (IV *vs* I-II-III) | 2.53 (1.02-6.26) | | **0.045** | 3.11 (1.14-8.51) | **0.03** | 1.87 (0.63-5.53) | 0.26 |
| **Site** |  | |  |  |  |  |  |
| Colorectal | 1.00 | |  | 1.00 |  | 1.00 |  |
| Ileum-duodenum-gallbladder | 1.12 (0.29-4.26) | | 0.87 | 1.12 (0.29-4.26) | 0.87 | 1.15 (0.28-4.83) | 0.85 |
| Gastroesophageal | 0.58 (0.19-1.83) | | 0.36 | 0.58 (0.19-1.83) | 0.36 | 0.31 (0.09-1.10) | 0.07 |
| Pancreas | 0.62 (0.23-1.67) | | 0.34 | 0.62 (0.23-1.67) | 0.34 | 0.23 (0.06-0.81) | **0.02** |
| **Histology** (≥55 *vs* <55) | 1.99 (0.85-4.67) | | 0.11 | 2.10 (0.88-5.02) | 0.10 | 3.02 (1.03-8.87) | **0.04** |
| **Therapy** |  | |  |  |  |  |  |
| None | 1.00 | |  | 1.00 |  | 1.00 |  |
| Others/SSA | 1.76 (0.19-15.96) | | 0.62 | 5.87 (0.70-49.22) | 0.10 | 5.36 (0.40-71.85) | 0.21 |
| Chemotherapy | 2.68 (0.35-20.31) | | 0.34 | 5.19 (0.42-64.88) | 0.20 | 2.81 (0.30-26.53) | 0.37 |
| **IHC p53** (Absent or overexpressed *vs* heterogeneous) | 2.13 (0.87-5.22) | | 0.10 | 3.09 (0.91-10.44) | 0.07 |  |  |
| **IHC Rb1** (Present *vs* Absent) | 0.37 (0.15-0.92) | | **0.03** | 0.35 (0.12-1.06) | 0.06 |  |  |
| **IHC SSTR-2A** (Present 2-3 *vs* Absent 0-1) | 1.60 (0.71-3.60) | | 0.25 | 2.23 (0.81-6.11) | 0.12 |  |  |
| **TMB** (>10 *vs* <10) | 0.88 (0.31-2.45) | | 0.80 | 1.05 (0.36-3.05) | 0.94 |  |  |
| ***TP53*** (Mut *vs* WT) | 2.72 (1.14-6.50) | | **0.02** | 2.60 (0.96-7.03) | 0.06 |  |  |
| ***APC*** (Mut *vs* WT) | 3.60 (1.41-9.21) | | **0.008** | 3.43 (1.06-11.12) | **0.04** |  |  |
| ***KRAS*** (Mut *vs* WT) | 3.26 (1.11-9.60) | | **0.03** | 2.94 (0.88-9.81) | 0.08 |  |  |

Abbreviation: HR, hazard ratio; CI, confidence interval; IHC, immunohistochemistry; p53, tumor suppressor p53; Rb1, retinoblastoma-associated protein; SSTR-2A, somatostatin receptor 2A; TBM, tumor mutational burden; *TP53*, tumor protein 53 gene; WT, wild type; *APC*, adenomatous polyposis coli gene; *KRAS*, Kirsten rat sarcoma virus gene.

**Supplementary Figure S1.** An expression-based molecular map was developed using UMAP method to understand the topological relationships between samples. A clear separation was observed between GEP-NET G3 and GEP-NEC with Ki-67 ≥55% samples while GEP-NEC with Ki-67 <55% samples were differently distributed between two separated groups.

**Supplementary Figure S2.** (**A**) Differential gene expression analysis of 8 Non-pancreatic neuroendocrine tumors (NETs) G3 and 20 Non-pancreatic neuroendocrine carcinomas (NECs) . The expression values of the 450 genes identified are arranged in rows. (**B**) Volcano plot depicting differentially expressed genes in Non-pancreatic NETs G3 and *vs* Non-pancreatic NECs. (**C**) Representation of up- and down-regulated pathways in Non-pancreatic NETs G3 and Non-pancreatic GEP NECs. Color legend: red and blue indicate high and low expression, respectively.


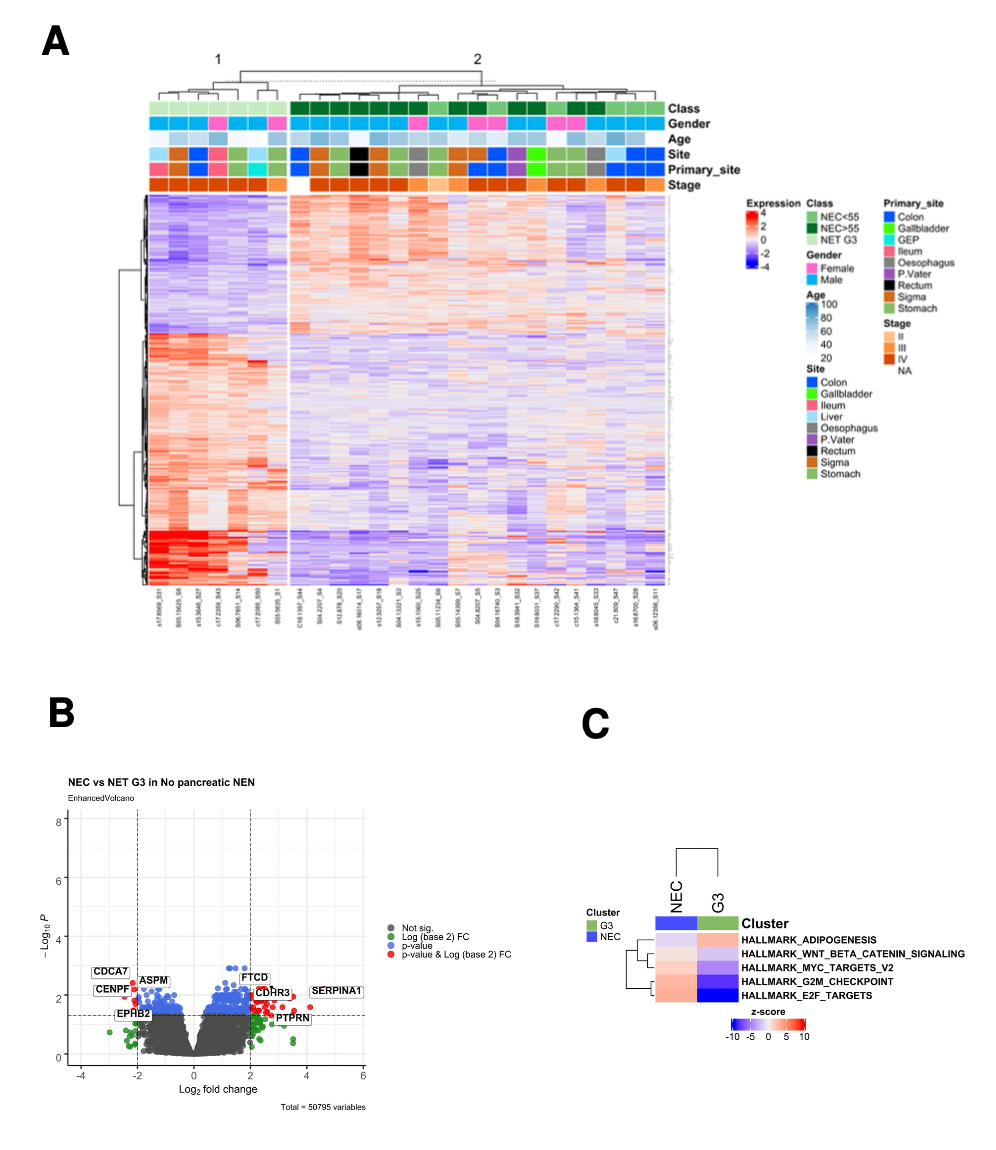


**Supplementary Figure S3.** Differential gene expression analysis of immune-related genes and immune microenvironment, TMB and MSI status, and differential expression of CTLA4 blockade signature (below) of 48 high-grade neuroendocrine neoplasms. Color legend: red and blue indicate high and low expression, respectively. Abbreviation: TMB, tumor mutational burden; MSI, microsatellite instability; CTLA4, cytotoxic T-lymphocyte antigen 4.


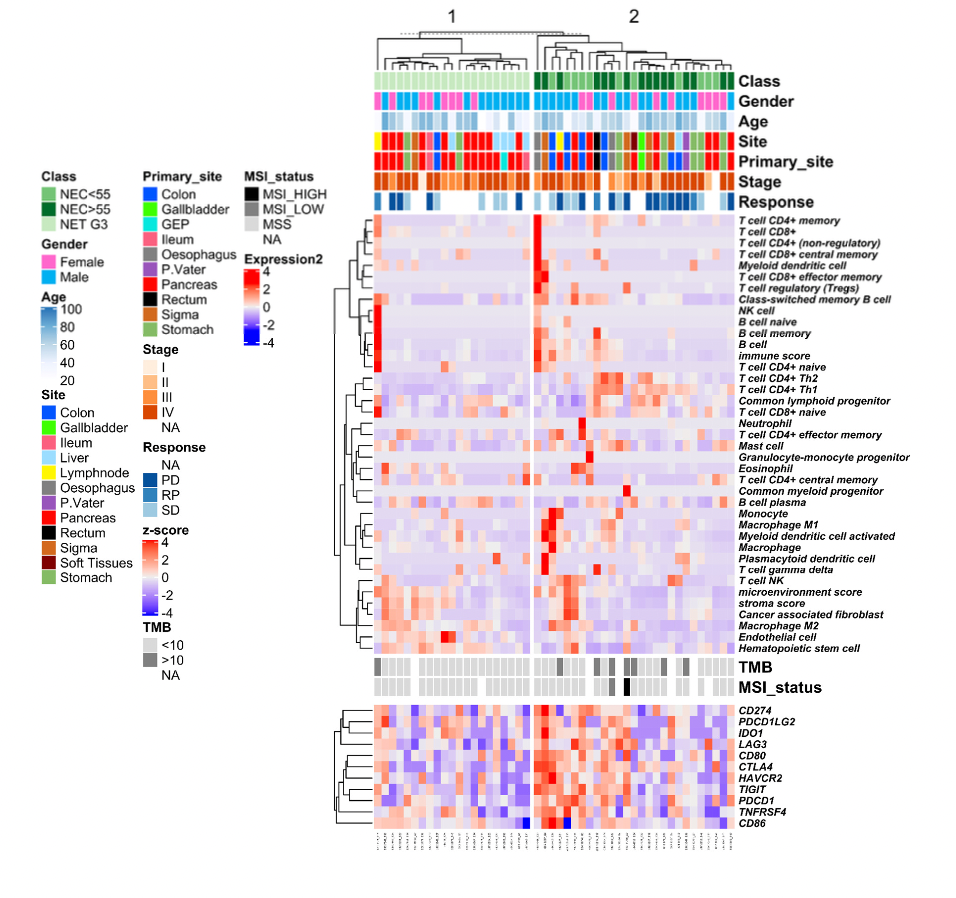


**Supplementary Figure S4.** (**A**) Supervised differential pathway expression analysis and (**B**) oncogene-selected expression analysis between tumor samples from patients with progressive disease (n=10) *vs* stable disease (n=7) following first-line chemotherapy. Color legend: red and blue indicate high and low expression, respectively.


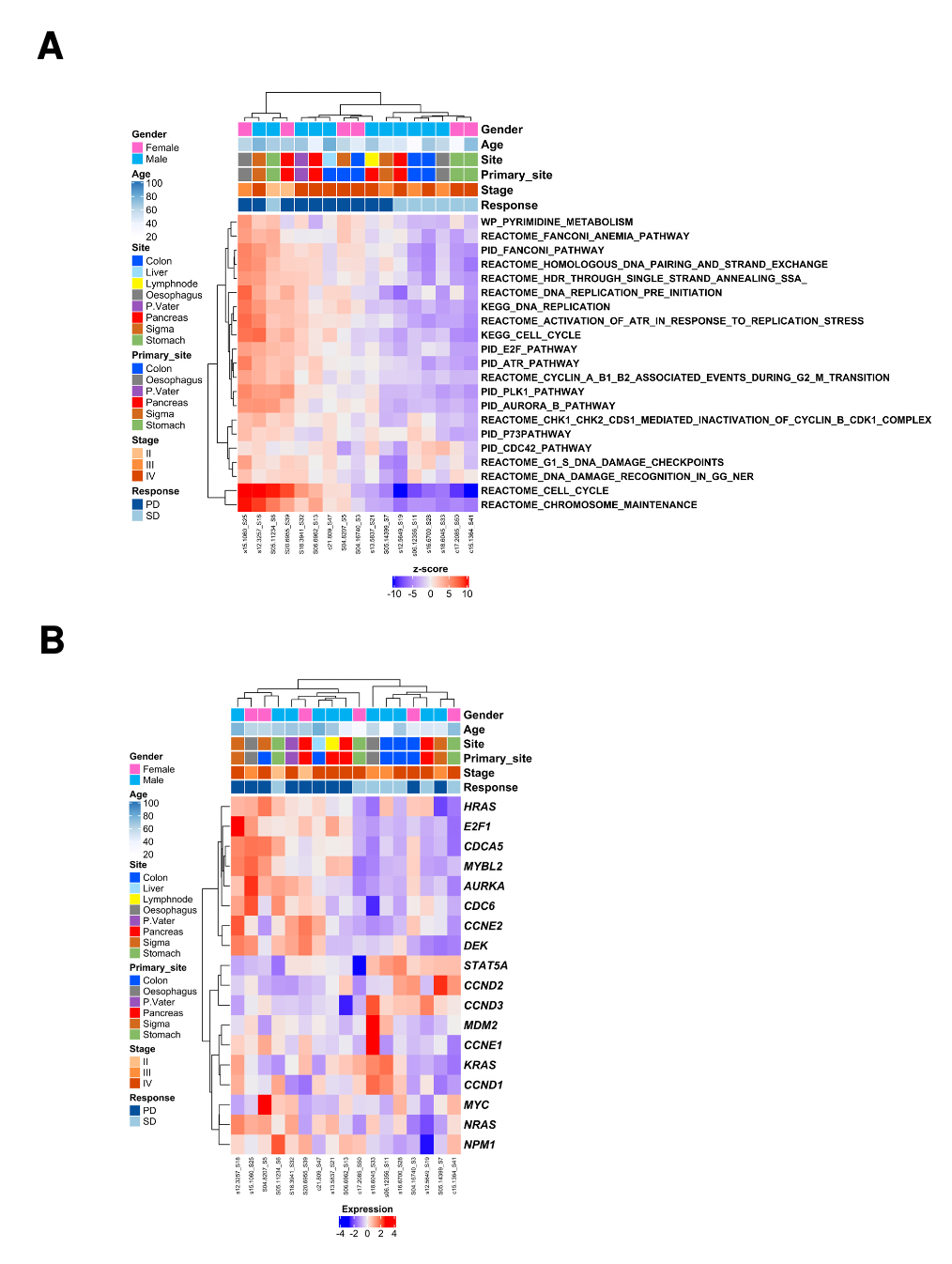


**Supplementary Figure S5.** Overall Survival (OS) of neuroendocrine tumors (NETs) G3 according to (**A**) p53 and (**B**) SSTR-2A expression.

**Supplementary Figure S6.** Overall Survival (OS) of neuroendocrine carcinomas (NECs) according to (**A**) *TP53*, (**B**) *APC,* and (**C**) *KRAS* mutational status.

**Supplementary Figure S7.** Overall Survival (OS) of neuroendocrine carcinomas (NECs) according to (**A**) p53 (**B**) Rb1*,* and (**C**) SSTR-2A expression status.
